# Correlates of repeat pregnancies among adolescent girls and young women in sub-Saharan Africa

**DOI:** 10.1186/s12884-023-05361-7

**Published:** 2023-02-03

**Authors:** Bright Opoku Ahinkorah, Richard Gyan Aboagye, Joshua Okyere, Abdul-Aziz Seidu, Eugene Budu, Sanni Yaya

**Affiliations:** 1 School of Public Health, Faculty of Health, University of Technology Sydney, Sydney, Australia; 2 Department of Family and Community Health, Fred N. Binka School of Public Health, University of Health and Allied Sciences, Hohoe, Ghana; 3 Department of Nursing, College of Health Sciences, Kwame Nkrumah University of Science and Technology, Kumasi, Ghana; 4 Department of Population and Health, University of Cape Coast, Cape Coast, Ghana; 5 Centre for Gender and Advocacy, Takoradi Technical University, Takoradi, Ghana; 6 College of Public Health, Medical and Veterinary Sciences, James Cook University, Townsville, Australia; 7 Korle Bu Teaching Hospital, P. O. Box, 77, Accra, Ghana; 8 School of International Development and Global Studies, University of Ottawa, Ottawa, Canada; 9 The George Institute for Global Health, Imperial College London, London, UK

**Keywords:** Adolescent girls, Repeat pregnancy, Sub-Saharan Africa, Young women

## Abstract

**Background:**

Adolescent girls and young women are vulnerable populations who are at risk of several adverse sexual and reproductive health outcomes, including unintended pregnancies, sexually transmitted infections, unsafe abortions, and death from pregnancy-related complications. In this study, we examined the correlates of repeat pregnancies among adolescent girls and young women in sub-Saharan Africa (SSA).

**Methods:**

We extracted data from the most recent Demographic and Health Surveys (DHS) of 31 countries in SSA. Countries whose surveys were conducted from 2010 to 2020 were included in the study. A total of 108,572 adolescent girls and young women (15–24 years) were included in the study. We used a multilevel mixed-effect binary logistic regression analysis to examine the correlates of repeat pregnancies among adolescent girls and young women in SSA.

**Results:**

We found that adolescent girls and young women aged 20–24 [aOR = 2.36; 95%CI = 2.22, 2.51], those married [aOR = 7.52; 95%CI = 6.81, 8.30], living with a partner [aOR = 7.51; 95%CI = 6.87, 8.21], and those who had sexual intercourse before age 20 [aOR = 1.41; 95%CI = 1.33, 1.51] had higher odds of experiencing repeat pregnancies compared to those aged 15–19, those never in a union, those whose first sexual intercourse occurred at age 20 and above, respectively. Respondents exposed to listening to radio [aOR = 1.12; 95%CI = 1.06, 1.18] and those who justified intimate partner violence [aOR=1.13; 95%CI = 1.07, 1.19] had higher odds of experiencing repeat pregnancies compared to those who never listened to radio and those who did not justify intimate partner violence, respectively. Young women who had attained secondary or higher educational level [aOR = 0.83; 95%CI = 0.78, 0.90], those exposed to reading newspaper or magazine [aOR = 0.90; 95%CI = 0.82, 0.98], those residing in rural areas [aOR = 0.92; 95%CI = 0.86, 0.98], and those belonging to the richer [aOR = 0.87; 95%CI = 0.80, 0.95] and richest [aOR = 0.68; 95%CI = 0.61, 0.76] wealth quintile were less likely to experience repeat pregnancies.

**Conclusion:**

The correlates of repeat pregnancies include age, age at first sexual intercourse, marital status, exposure to media, justification of intimate partner violence, wealth index, educational attainment, and place of residence. The findings underscore the need for governments and policymakers in SSA to implement policies that target the most at-risk groups: those with no formal education, the poor, and adolescent girls. Our findings also highlight the need to strengthen advocacy against the justification of intimate partner violence and intensify girl-child education.

## Background

Adolescent girls and young women (AGYW) are vulnerable populations who are at risk of several adverse sexual and reproductive health (SRH) outcomes including unintended pregnancies, sexually transmitted infections (STIs), unsafe abortions, and death from pregnancy-related complications [1–3]. As such, there have been various policy frameworks and global commitments to reduce AGYW SRH vulnerabilities. Notable among these policy frameworks include the Programme of Action which was ratified during the 1994 International Conference on Population and Development (ICPD); other frameworks include the ended Millennium Development Goals (MDGs), and the 2015 affirmed 17 Sustainable Development Goals (SDGs) [3–5]. These efforts have culminated in a reduction of birth among AGYW from 65 births per 1000 women in 1990 to 47 births per 1000 women in 2015 [6].

Nonetheless, available evidence indicates that nearly 21 million adolescent girls get pregnant worldwide each year [7]. The burden of these pregnancies is endemic across sub-Saharan African countries [8]. Relatedly, the issue of repeat pregnancy stares glaringly in the face of the high pregnancy rate among AGYW. Thus, making it an important public health and social problem [9]. Repeat pregnancy is defined as a second pregnancy or additional pregnancies to AGYW within 12–24 months of the previous pregnancy [9]. In the United States of America, the rate of repeat pregnancies ranges between 12%—49% [10]. In South Africa, the rate of repeat pregnancy stands at 17.3% [9, 11] whereas in Uganda, repeat pregnancy among AGYW within 24 months stands at 73% [12].

Repeat pregnancy is a serious public health and social problem because of its implications on the overall health and wellbeing of AGYW. Previous studies have shown that AGYW’s experience of repeat pregnancy has significant adverse implications on medical, educational, socioeconomic, and parenting outcomes [9, 13]. For instance, Maravilla et al. [14] argue that repeat pregnancy may result in higher physiological and behavioral health consequences, such as pregnancy complications, psychological discomfort, and economic reliance owing to their inability to complete school education. These adverse implications of repeat pregnancy on AGYW's health and wellbeing underscore the need to unearth and understand its correlates within the sub-Saharan Africa (SSA).

There have been previous studies conducted in individual sub-Saharan African countries that have examined the correlates of repeat pregnancy among AGYW. For instance, studies conducted in South Africa [9] and Uganda [12] have shown that the correlates of repeat pregnancy include partner’s level of education, place of residence, age at first union, marriage-to-birth interval, history of spontaneous abortion, previous contraceptive use, emotional support, and AGYW’s level of education. However, the dynamics and nuances from a subregional perspective have not been explored by previous studies. To the best of our knowledge after an extensive literature search, there is no study that has investigated the correlates of repeat pregnancy among AGYW across sub-Saharan African countries. This presents a significant gap on the correlates of repeat pregnancy among AGYW in SSA. The study examines the prevalence and correlates of repeat pregnancy among AGYW in SSA using data from the most recent nationally representative dataset.

## Methods

### Data source and study design

We extracted data from the most recent Demographic and Health Surveys (DHS) of 31 countries in SSA. Countries whose surveys were conducted from 2010 to 2020 were included in the study. DHS is a comparative nationwide survey conducted across over 90 countries with the aim of enhancing the collection, analysis, and dissemination of demographic, health, and nutrition data to increase the usefulness of these data for program administration, planning, and policymaking [15]. The DHS employed a cross-sectional design in collecting the data from the respondents [16]. Respondents were sampled for the survey using a two-stage cluster sampling technique. Detailed sampling methodology has been highlighted in literature [16, 17]. Pretested and validated structured questionnaires were used to collect the data from the respondents with the aid of well-trained data collectors. The total sample used was 108,572 AGYW (Table 1). The sample size was obtained by considering countries with DHS data published from 2010 to 2020; countries who had complete cases on all the variables of interest in this study; and AGYW aged 15–24. We drafted this paper with reference to the Strengthening the Reporting of Observational Studies in Epidemiology (STROBE) guidelines [18].

**Table 1:** Sample distribution

Country	Survey Year	Weighted Frequency	Weighted Percentage
1. Angola	2015–16	3,406	3.1
2. Burkina Faso	2010	4,201	3.9
3. Benin	2018	3,856	3.6
4. Burundi	2016–17	4,314	4.0
5. DR Congo	2013–14	4,505	4.1
6. Cote d’Ivoire	2011–12	2,435	2.2
7. Cameroon	2018	3,405	3.1
8. Ethiopia	2016	3,525	3.2
9. Gabon	2012	1,984	1.8
10. Ghana	2014	2,251	2.1
11. Gambia	2019–20	2,556	2.4
12. Guinea	2018	2,682	2.5
13. Kenya	2014	7,763	7.2
14. Comoros	2012	1,230	1.1
15. Liberia	2019–20	1,932	1.8
16. Lesotho	2014	1,570	1.4
17. Mali	2018	2,540	2.3
18. Malawi	2015–16	6,048	5.6
19. Nigeria	2018	10,114	9.3
20. Niger	2012	2,874	2.6
21. Namibia	2013	2,447	2.3
22. Rwanda	2019–20	3,573	3.3
23. Sierra Leone	2019	3,760	3.5
24. Senegal	2010–11	3,354	3.1
25. Chad	2014–15	4,387	4.0
26. Togo	2013–14	2,318	2.1
27. Tanzania	2015–16	3,445	3.2
28. Uganda	2016	4,475	4.1
29. South Africa	2016	2,021	1.9
30. Zambia	2018	3,260	3.0
31. Zimbabwe	2015	2,341	2.2
All countries	2010–2020	108,572	100.0

The third-party data underlying the results presented in the study are available from The DHS Program. Users can register on The DHS Program website (https://dhsprogram.com/). Once registered, interested researchers can request access to the DHS datasets. The datasets generated and/or analyzed during the current study are available in http://dhsprogram.com/data/available-datasets.cfm.

### Variables

Repeat pregnancies was the outcome variable in the study. We assessed repeat pregnancies using three variables (ever given birth [no/yes], currently pregnant [no/yes], and ever terminated pregnancy [no/yes]) based on literature [12]. AGYW who reported “yes” in at least two of the three variables were categorized as having repeat pregnancies. Those whose response options were “no” in all the three variables or responded “yes” to one of the variables were grouped as not experienced repeat pregnancies.

Twelve explanatory variables were considered for this this study and they were selected based on their availability in the DHS dataset and from previous studies [12, 19, 20]. We segregated the variables into individual and contextual level variables respectively. Aside the place of residence and geographical subregions, the remaining variables were considered as individual-level variables. We utilized the existing coding as found in the DHS for current working status (no/yes), wealth index (poorest, poorer, middle, richer, and richest), and place of residence (urban/rural). For the remaining variables, we recoded them as follows; age of AGYW (“15–19” and “20–24”); level of education (“no education”, “primary”, and “secondary or higher”); marital status (“never in union”, “married”, “cohabiting”, and “previously married”); age at first sexual intercourse (“ ≤ 19 years” and “20 years and above”); exposure to listening to radio (“no” and “yes”); exposure to reading newspaper or magazine (“no” and “yes”); exposure to watching television (“no” and “yes”); justification of intimate partner violence (“no” and “yes”) and geographical subregions (“Eastern”, “Central”, “Southern”, and “Western”). The 31 countries were used to create the geographical subregions based on their locations in SSA.

### Statistical analyses

**Fig. 1:**
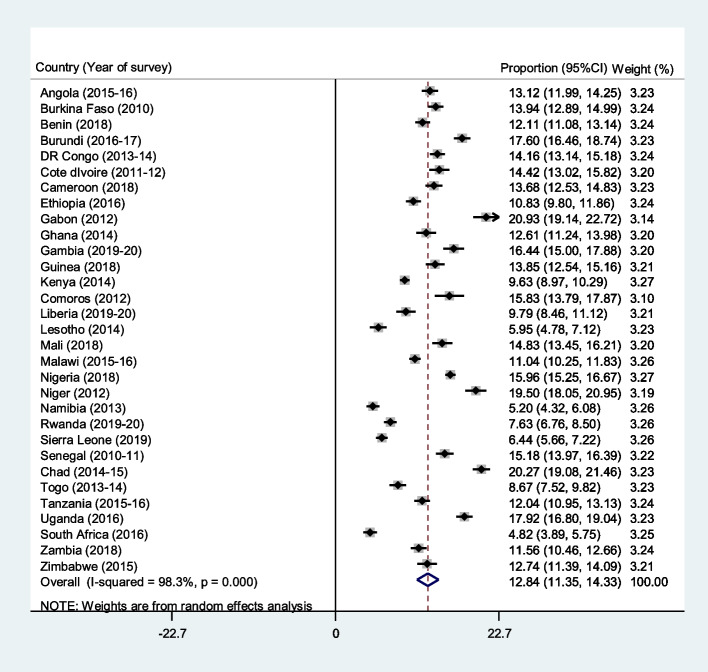
Forest plot showing the prevalence of repeat pregnancies among the adolescent girls and young women in sub-Saharan Africa

We utilized descriptive and inferential statistics to determine the prevalence of repeat pregnancies and its associated factors, respectively. Stata software version 16.0 was used for the analyses. We employed a forest plot to present the results of the prevalence of repeat pregnancies (Fig. 1). Later, cross-tabulation was performed to determine the distribution of repeat pregnancies across the explanatory variables (Table 2). We adopted the Pearson chi-square test of independence to examine the explanatory variables significantly associated with repeat pregnancies (Table 2). All the variables with p-values less than 0.05 were placed in the regression model. Additionally, we conducted a multi-collinearity test to ascertain the level of collinearity among the explanatory variables using the variance inflation factor (VIF). The results showed that the minimum, maximum, and mean VIF were 1.01, 2.39, and 1.54, respectively. Based on the results for the VIF, there was no evidence of collinearity among the variables included in the regression analysis. We used a multilevel mixed-effect binary logistic regression analysis to examine the predictors of repeat pregnancy among the AGYW in SSA using four models (Model O-III). We placed only the repeat pregnancy in the first model (Model O). Model I and II consisted of the individual and contextual level variables respectively. The last model (Model III) contained all the explanatory variables. The results were segregated into fixed and random effects. In the fixed-effect model, adjusted odds ratio (aOR) with their respective 95% confidence intervals (CIs) were used to present the results. Hence, the fixed-effect model measures the relationship between the explanatory variables and repeat pregnancy. On the other hand, the random effects model measured the variation in the repeat pregnancy based on the primary sampling unit (PSU) (measured by Intra-Cluster Correlation Coefficient [ICC]). The Akaike's Information Criterion (AIC) was used to evaluate model fitness, or how well various models fitted the data. The multilevel regression models were run with Stata's "melogit" function. We addressed potential bias by ensuring that data management and analyses were verified by all the authors. The "svyset" command was used to adjust for disproportionate sampling and non-response, and weighting was carried out to take into consideration the complex makeup of DHS data. Missing data were handled using complete cases through listwise deletion.

**Table 2:** Distribution of repeat pregnancies across the explanatory variables

**Variable**			**Repeat Pregnancies**		
	**Weighted frequency (N)**	**Weighted percentage (%)**	**No [95% CI]**	**Yes [95% CI]**	***P*-value**
**Women’s age (years)**					
15–19	38,021	35.0	93.4 [93.0, 93.7]	6.6 [6.3, 7.0]	< 0.001
20–24	70,551	65.0	83.4 [83.0, 83.7]	16.6 [16.3, 17.0]	
**Level of education**					
No education	27,508	25.3	82.0 [81.4, 82.5]	18.0 [17.5, 18.6]	< 0.001
Primary	34,333	31.6	85.0 [84.5, 85.5]	15.0 [14.5, 15.5]	
Secondary or higher	46,731	43.0	91.1 [90.7, 91.4]	8.9 [8.6, 9.3]	
**Marital status**					
Never in union	37,629	34.7	97.5 [97.3, 97.7]	2.5 [2.3, 2.7]	< 0.001
Married	51,750	47.7	81.0 [80.6, 81.4]	19.0 [18.6, 19.4]	
Living with partner	13,478	12.4	79.3 [78.3, 80.2]	20.7 [19.8, 21.7]	
Widowed	337	0.3	88.3 [83.0, 92.2]	11.7 [7.8, 17.0]	
Divorced	1,742	1.6	90.9 [89.1, 92.4]	9.1 [7.6, 10.9]	
Separated	3,635	3.3	86.9 [85.5, 88.3]	13.1 [11.7, 14.5]	
**Current working status**					
No	55,600	51.2	88.2 [87.8, 88.5]	11.8 [11.5, 12.2]	< 0.001
Yes	52,972	48.8	85.5 [85.1, 85.9]	14.5 [14.1, 14.9]	
**Age at first sexual intercourse**					
20 years and above	21,002	19.3	85.9 [85.2, 86.5]	14.1 [13.5, 14.8]	< 0.001
Below 20 years	87,570	80.7	87.1 [86.8, 87.4]	12.9 [12.6, 13.2]	
**Exposed to watching television**					
No	60,105	55.4	85.1 [84.7, 85.5]	14.9 [14.5, 15.3]	< 0.001
Yes	48,467	44.6	89.1 [88.7, 89.5]	10.9 [10.5, 11.3]	
**Exposed to listening to radio**					
No	45,056	41.5	86.0 [85.6, 86.4]	14.0 [13.6, 14.4]	< 0.001
Yes	63,516	58.5	87.5 [87.1, 87.8]	12.5 [12.2, 12.9]	
**Exposed to reading newspaper or magazine**					
No	85,113	78.4	85.6 [85.3,85.9]	14.4 [14.1,14.7]	< 0.001
Yes	23,459	21.6	91.6 [91.0, 92.1]	8.4 [7.9, 9.0]	
**Justified intimate partner violence**					
No	56,581	52.1	88.5 [88.2, 88.9]	11.5 [11.1, 11.8]	< 0.001
Yes	51,991	47.9	85.0 [84.6, 85.4]	15.0 [14.6, 15.4]	
**Wealth index**					
Poorest	20,087	18.5	84.0 [83.4, 84.6]	16.0 [15.4, 16.6]	< 0.001
Poorer	21,895	20.2	84.2 [83.6, 84.8]	15.8 [15.2, 16.4]	
Middle	21,540	19.8	86.1 [85.5, 86.7]	13.9 [13.3, 14.5]	
Richer	22,519	20.7	88.0 [87.4, 88.6]	12.0 [11.4, 12.6]	
Richest	22,531	20.8	91.5 [91.0, 92.1]	8.5 [7.9, 9.0]	
**Place of residence**					
Urban	41,060	37.8	89.4 [89.0,89.9]	10.6 [10.1, 11.0]	< 0.001
Rural	67,512	62.2	85.3 [85.0,85.6]	14.7 [14.4, 15.0]	
**Subregions**					
Central Africa	17,686	16.3	83.9 [83.2, 84.5]	16.1 [15.5, 16.8]	< 0.001
West Africa	44,874	41.3	86.2 [85.8, 86.7]	13.8 [13.3, 14.2]	
East Africa	28,325	26.1	87.4 [86.7, 88.0]	12.6 [12.0, 13.3]	
Southern Africa	17,686	16.3	90.6 [90.0, 91.1]	9.4 [8.9, 10.0]	

### Ethical consideration

Ethical approval was not sought for this study since we used a freely accessible data in the public domain. We obtained the permission to download and use the dataset for publication from the MEASURE DHS. We complied with the ethical guidelines accompanying the use of secondary dataset for publication. The detailed outline of the ethical guidelines can be accessed at http://goo.gl/ny8T6X.

## Results

### Prevalence of repeat pregnancies among adolescent girls and young women in sub-Saharan Africa

Figure 1 shows sample distribution and prevalence of repeat pregnancy across the 31 countries studied. The pooled prevalence of repeat pregnancies among the AGYW was 12.84%. The prevalence of repeat pregnancies was highest in Gabon (20.93%) and lowest in South Africa (4.82%).  The distribution of repeat pregnancies across the explanatory variables are also showed in Table 2. Young women aged 20–24 (16.6%), those with no formal education (18.0%), those living with their partner (20.7%), and those working (14.5%) had the highest prevalence of repeat pregnancy. Additionally, AGYW in the poorest wealth quintile (16.0%), those in rural (14.7%), and Central Africa (16.1%) had the highest prevalence of repeat pregnancy.

**Table 3:** Mixed effect analysis of factors associated with repeat pregnancies among adolescent girls and young women in sub-Saharan Africa

Variables	Model O	Model I aOR [95% CI]	Model II aOR [95% CI]	Model III aOR [95% CI]
**Fixed effects results**				
**Women’s age (years)**				
15–19		1.00		1.00
20–24		2.30^***^ [2.16, 2.44]		2.36^***^ [2.22, 2.51]
**Level of education**				
No education		1.00		1.00
Primary		0.98 [0.93, 1.04]		1.04 [0.97, 1.11]
Secondary or higher		0.83^***^ [0.78, 0.89]		0.83^***^ [0.78, 0.90]
**Marital status**				
Never in union		1.00		1.00
Married		7.17^***^ [6.56, 7.84]		7.51^***^ [6.87, 8.21]
Cohabiting		7.82^***^ [7.08, 8.63]		7.52^***^ [6.81, 8.30]
Widowed		3.62^***^ [2.32, 5.63]		3.67^***^ [2.35, 5.74]
Divorced		2.96^***^ [2.37, 3.70]		3.17^***^ [2.54, 3.96]
Separated		4.42^***^ [3.78, 5.17]		4.42^***^ [3.78, 5.17]
**Current working status**				
No		1.00		1.00
Yes		1.05^*^ [1.00, 1.11]		1.05 [1.00, 1.10]
**Age at first sexual intercourse**				
20 years and above		1.00		1.00
Below 20 years		1.30^***^ [1.23, 1.38]		1.41^***^ [1.33, 1.51]
**Exposed to watching television**				
No		1.00		1.00
Yes		1.08^**^ [1.02, 1.15]		1.03 [0.97, 1.09]
**Exposed to listening to radio**				
No		1.00		1.00
Yes		1.04 [0.98, 1.09]		1.12^***^ [1.06, 1.18]
**Exposed to reading newspaper or magazine**				
No		1.00		1.00
Yes		0.88^**^ [0.81, 0.96]		0.90^*^ [0.82, 0.98]
**Justified intimate partner violence**				
No		1.00		1.00
Yes		1.16^***^ [1.10, 1.22]		1.13^***^ [1.07, 1.19]
**Wealth index**				
Poorest		1.00		1.00
Poorer		1.02 [0.95, 1.10]		1.00 [0.94,1.08]
Middle		0.97 [0.91, 1.05]		0.94 [0.88, 1.01]
Richer		0.91^*^ [0.84, 0.99]		0.87^**^ [0.80, 0.95]
Richest		0.73^***^ [0.66, 0.80]		0.68^***^ [0.61, 0.76]
**Place of residence**				
Urban			1.00	1.00
Rural			1.54^***^ [1.46, 1.63]	0.92^*^ [0.86, 0.98]
**Subregions**				
Central Africa			1.00	1.00
West Africa			0.81^***^ [0.76, 0.86]	0.66^***^ [0.62, 0.71]
East Africa			0.71^***^ [0.65, 0.77]	0.58^***^ [0.54, 0.64]
Southern Africa			0.50^***^ [0.46, 0.54]	0.53^***^ [0.48, 0.58]
**Random effects results**				
PSU variance (95% CI)	1.095 [0.892, 1.344]	0.968 [0.780, 1.201]	1.088 [0.883, 1.340]	0.952 [0.766, 1.182]
ICC	0.250	0.227	0.248	0.224
Wald chi-square	Reference	4049.26 (< 0.001)	467.14 (< 0.001)	4444.89 (< 0.001)
**Model fitness**				
Log-likelihood	-171,977.29	-153,783.65	-170,275.34	-152,951.47
AIC	343,958.6	307,607.3	340,562.7	305,950.9
BIC	343,977.8	307,799.2	340,620.2	306,181.2
N	108,572	108,572	108,572	108,572
Number of clusters	1,591	1,591	1,591	1,591

*aOR* adjusted odds ratios, *CI* Confidence Interval,^*^p < 0.05, ^**^p < 0.01, ^***^p < 0.001; 1=Reference category; *PSU* Primary Sampling Unit, *ICC* Intra-Class Correlation, *AIC* Akaike’s Information Criterion

### Mixed effect analysis of factors associated with repeat pregnancies among adolescent girls and young women in sub-Saharan Africa

#### Fixed effects results

Table 3 shows the mixed effect analysis of factors associated with repeat pregnancy among AGYW in SSA. We found that young women aged 20–24 [aOR = 2.36; 95%CI = 2.22, 2.51], those married [aOR = 7.52; 95%CI = 6.81, 8.30], those living with a partner [aOR = 7.51; 95%CI = 6.87, 8.21], and those who had sexual intercourse before age 20 [aOR = 1.41; 95%CI = 1.33, 1.51] had higher odds of experiencing repeat pregnancies compared to those aged 15–19, those never in a union, those whose first sexual intercourse occurred at age 20 and above respectively. Respondents exposed to listening to radio [aOR = 1.12; 95%CI = 1.06, 1.18] and those who justified intimate partner violence [aOR=1.13; 95%CI = 1.07, 1.19] had higher odds of experiencing repeat pregnancies compared to those who never listen to radio and those who did not justify intimate partner violence, respectively. Young women who had attained secondary or higher educational level [aOR = 0.83; 95%CI = 0.78, 0.90], those exposed to reading newspaper or magazine [aOR = 0.90; 95%CI = 0.82, 0.98], those residing in rural areas [aOR = 0.92; 95%CI = 0.86, 0.98], and those belonging to the richer [aOR = 0.87; 95%CI = 0.80, 0.95] and richest [aOR = 0.68; 95%CI = 0.61, 0.76] wealth quintile were less likely to experience repeat pregnancy. At the subregional level, compared to young women from Central Africa, those residing in Western, Eastern, and Southern had lower odds of experiencing repeat pregnancy.

#### Random effects results

The results of the random effects are also presented in Table 3. It was shown that the ICC value for the null model was 0.25 which shows that about 25% of the variation in repeat pregnancy is attributed to variation between clusters. This variation reduced slightly to 23% in the individual level model and increased slightly to 25% in the contextual variables model and reduced a little again to 22% in the complete model. Model III which is the complete model with individual, household and community level variables had a lower AIC value of 305,950.9 compared to the other models which show that it is the model of best fit.

## Discussion

The present study sought to examine the prevalence and correlates of repeat pregnancy among AGYW in SSA. Our findings show that 12.84% of AGYW in SSA had experienced repeat pregnancy. Despite the seeming lower prevalence of repeat pregnancy across sub-Saharan African countries, the prevalence varies between countries. For instance, Gabon (20.93%) reported the highest prevalence of repeat pregnancy whereas South Africa (4.82%) reported the lowest prevalence. Thus, suggesting the existence of heterogeneity in terms of the associated or risk factors across SSA. Consistent with findings from previous studies [14, 21, 22], our study showed that the correlates of repeat pregnancy among AGYW in SSA included age, sexual debut, marital status, exposure to media, justification of intimate partner violence, wealth, educational attainment, and place of residence.

Concerning age, our findings revealed that young women aged 20-24 years were more likely to have repeat pregnancies. Similar findings have been reported in South Ethiopia where it was revealed that younger age was strongly correlated with the risk of having repeat pregnancies [22]. Perhaps, the result could be explained from the perspective that during the period of adolescence, the individual is often naive and curious and tend to have the exuberance to experiment with sex [23]. Hence, raising their risk of having additional pregnancies before age 24. Often, adolescent girls who get pregnant for the first time drop out of school and struggle with getting the resources to cater for their needs and that of their children [24]. This situation may unduly influence young women to engage in transactional sex that may subsequently exacerbate their risk of repeat pregnancy. Our finding could be from the point that in most sub-Saharan African countries, AGYW are catered for by their parents. Therefore, in the event of a first pregnancy, parents, out of frustration may unofficially hand over their child to the person who impregnated her [25, 26]. Such practices may foster a higher likelihood of repeat pregnancy among young women.

Our study revealed that educational attainment was significantly associated with repeat pregnancy among AGYW in SSA. The odds of having repeat pregnancy was significantly lower among those with secondary or higher education compared to those with no formal education. The result is corroborated by evidence from previous studies that have found higher educational attainment to be associated with a lower risk of repeat pregnancies [21, 22]. We postulate that the association between higher educational attainment and lower risk of repeat pregnancy could be explained from the perspective that getting formal education delays marriage, which is the traditionally acceptable context for procreation in SSA [27, 28]. Hence, reducing AGYW with higher education’s risk of repeat pregnancy. Another plausible explanation could be that AGYW with higher education are well-informed about the risk of repeat pregnancy, and therefore, are more likely to autonomously make healthcare decisions including negotiating for safer sex [29].

We found marital status to be significantly associated with the risk of having repeat pregnancies. Specifically, AGYW who were married or cohabiting with their partner were more likely to have repeat pregnancies compared to their counterparts who have never married. One of the plausible reasons for this finding could be that marriage and cohabitation create some sort of legitimacy to sex within the SSA context, thereby increasing AGYW’s desire for more children [30]. Another possible explanation for this could be the potential interplay of power relations. Married and cohabiting AGYW may lack the capacity to negotiate for safer sex practices such as the use of condoms and other contraceptives [29]. This may exacerbate their likelihood of experiencing repeat pregnancy.

The present study revealed that belonging to a higher wealth index was significantly associated with a lower risk of repeat pregnancy. Similar findings have been reported in a quantitative study conducted in the Philippines that suggests a positive association between wealth index and the risk of repeat pregnancies [14]. The pathway could be that unlike those from the poorest wealth index who tend to marry early and place much value on children as assets, AGYW from the richer wealth index marry late, and have minimal value on children as assets [31]. Also, our study revealed that women who justified intimate partner violence were more likely to have repeat pregnancies. This is not surprising because the justification of violence by an intimate partner is an indicator of weak or absent empowerment and autonomy. Thus, such individuals are likely not to be able to negotiate for safer sex and therefore, exacerbate their risk of repeat pregnancy [29].

Lastly, at the sub-regional level, compared to the Central part of SSA, AGYW in the other sub-regions had a significantly lower risk of repeat pregnancies. We are uncertain of the reasons accounting for this finding. However, our finding points to the fact that there is substantial heterogeneity across the sub-region that could be potentially explored in future studies.

### Policy implications

Overall, our findings call for the need to strengthen existing policies on adolescent pregnancy in SSA. Reference to the scoping review of national policies on prevention of adolescent pregnancy in anglophone SSA can help identify some of these effective policies which can be implemented in countries with high prevalence of repeat pregnancies among adolescents [32]. Our findings about the association between educational attainment and repeat pregnancy is a wake-up call to all governments and policymakers in SSA to invest in formal education for AGYW. Also, the result concerning wealth index and repeat pregnancy highlights a need for pro-poor interventions. Such interventions can contribute to reducing repeat pregnancies in SSA. Concerning our findings on the justification of intimate partner violence and the risk of repeat pregnancies, it is a call for all actors to intensify advocacy and empowerment of AGYW in SSA.

### Strengths and limitations

The study’s strength lies in the use of a nationally representative dataset, and also, in the analysis of the correlates of repeat pregnancy at the sub regional level. Nevertheless, there are some limitations that we acknowledge. First, we are unable to draw causal inferences due to the cross-sectional nature of DHS. Also, because we relied on secondary data, we are unable to assess how residual factors (e.g., cultural norms and expectations, perceived risks, perceived benefits, cues to action) correlate with the risk of repeat pregnancy across SSA. The variable on repeat pregnancy was self-reported and is thus prone to recall and social desirability bias. Another important limitation that must be considered is the variation in the survey years of the DHS data. Some of the datasets (i.e., Burkina Faso, Gabon, Ghana, Kenya, Comoros, Lesotho, Namibia, Niger, Senegal and Togo) were collected before the ratification and implementation of the SDGs which are the current blueprint to guide policies that will end repeat pregnancies. As such, observations from countries with older DHS dataset, although informative, may not be reflective of the current status quo.

## Conclusion

This study examined the correlates of repeat pregnancy among AGYW in SSA using data from the most recent DHS. We conclude that the correlates of repeat pregnancy include age, age at first sexual intercourse, marital status, exposure to media, justification of intimate partner violence, wealth index, and educational attainment. The findings underscore the need for governments and policymakers in SSA countries to implement policies that target the most at-risk groups: those with no formal education, the poor and adolescent girls. Our findings also highlight the need to strengthen advocacy against the justification of intimate partner violence and intensify girl-child education.

## Data Availability

The third-party data underlying the results presented in the study are available from The DHS Program. Users can register on The DHS Program website (https://dhsprogram.com/). Once registered, interested researchers can request access to the DHS datasets. The datasets generated and/or analyzed during the current study are available in http://dhsprogram.com/data/available-datasets.cfm.
